# PSEN1-selective gamma-secretase inhibition in combination with kinase or XPO-1 inhibitors effectively targets T cell acute lymphoblastic leukemia

**DOI:** 10.1186/s13045-021-01114-1

**Published:** 2021-06-24

**Authors:** Inge Govaerts, Cristina Prieto, Charlien Vandersmissen, Olga Gielen, Kris Jacobs, Sarah Provost, David Nittner, Johan Maertens, Nancy Boeckx, Kim De Keersmaecker, Heidi Segers, Jan Cools

**Affiliations:** 1 Center for Human Genetics, KU Leuven, Leuven, Belgium; 2 Center for Cancer Biology, VIB, Leuven, Belgium; 3 Leuvens Kanker Instituut (LKI), KU Leuven – UZ Leuven, Leuven, Belgium; 4 Department of Hematology, UZ Leuven, Leuven, Belgium; 5 Department of Microbiology, Immunology and Transplantation, KU Leuven, Leuven, Belgium; 6 Department of Laboratory Medicine, UZ Leuven, Leuven, Belgium; 7 Department of Oncology, KU Leuven, Leuven, Belgium; 8 Department of Pediatric Oncology, UZ Leuven, Leuven, Belgium

**Keywords:** Leukemia, Oncogenes, Targeted therapy, Mouse models, Gamma-secretase complex, Signaling, Nuclear export, Toxicity

## Abstract

**Background:**

T cell acute lymphoblastic leukemia (T-ALL) is a high-risk subtype that comprises 10–15% of childhood and 20–25% of adult ALL cases. Over 70% of T-ALL patients harbor activating mutations in the NOTCH1 signaling pathway and are predicted to be sensitive to gamma-secretase inhibitors. We have recently demonstrated that selective inhibition of PSEN1-containing gamma-secretase complexes can overcome the dose-limiting toxicity associated with broad gamma-secretase inhibitors. In this study, we developed combination treatment strategies with the PSEN1-selective gamma-secretase inhibitor MRK-560 and other targeted agents (kinase inhibitors ruxolitinib and imatinib; XPO-1 inhibitor KPT-8602/eltanexor) for the treatment of T-ALL.

**Methods:**

We treated T-ALL cell lines in vitro and T-ALL patient-derived xenograft (PDX) models in vivo with MRK-560 alone or in combination with other targeted inhibitors (ruxolitinib, imatinib or KPT-8602/eltanexor). We determined effects on proliferation of the cell lines and leukemia development and survival in the PDX models.

**Results:**

All NOTCH1-signaling-dependent T-ALL cell lines were sensitive to MRK-560 and its combination with ruxolitinib or imatinib in JAK1- or ABL1-dependent cell lines synergistically inhibited leukemia proliferation. We also observed strong synergy between MRK-560 and KPT-8602 (eltanexor) in all NOTCH1-dependent T-ALL cell lines. Such synergy was also observed in vivo in a variety of T-ALL PDX models with NOTCH1 or FBXW7 mutations. Combination treatment significantly reduced leukemic infiltration in vivo and resulted in a survival benefit when compared to single treatment groups*.* We did not observe weight loss or goblet cell hyperplasia in single drug or combination treated mice when compared to control.

**Conclusions:**

These data demonstrate that the antileukemic effect of PSEN1-selective gamma-secretase inhibition can be synergistically enhanced by the addition of other targeted inhibitors. The combination of MRK-560 with KPT-8602 is a highly effective treatment combination, which circumvents the need for the identification of additional mutations and provides a clear survival benefit in vivo. These promising preclinical data warrant further development of combination treatment strategies for T-ALL based on PSEN1-selective gamma-secretase inhibition.

**Supplementary Information:**

The online version contains supplementary material available at 10.1186/s13045-021-01114-1.

## Background

Acute lymphoblastic leukemia (ALL) is an aggressive hematological malignancy characterized by a rapid clonal expansion of immature lymphocytes that infiltrate the bone marrow. T cell acute lymphoblastic leukemia (T-ALL) comprises 10–15% of childhood and 20–25% of adult cases of ALL and is considered a high-risk subtype of this aggressive hematological malignancy [1]. Despite the increase in cure rates with intensified chemotherapy treatment, the prognosis remains poor for primary refractory or relapsed ALL and the likelihood of favorable outcome steeply declines with increasing age [2].

At diagnosis, T-ALL patients carry 10–20 genomic lesions, including coding mutations and structural variants, which can increase by twofold in adults or in case of relapse [3, 4]. T-ALL is typically characterized by ectopic expression of an oncogenic transcription factor (TLX1, TLX3, TAL1, NKX3-1) and loss of cell cycle regulators CDKN2A/CDKN2B. Other aberrations typically cause activation of signaling pathways, such as NOTCH1 signaling, the JAK-STAT pathway, RAS-MAPK signaling or the PI3K-AKT-mTOR pathway. Chromosomal rearrangements can lead to expression of fusion genes, such as the NUP214-ABL1 [5] or ETV6-ABL1 fusion kinases [6]. Finally, mutations can alter the function of epigenetic regulators and chromatin remodelers or influence RNA processing [7, 8].

Through sequencing efforts in the past decade, the genomic landscape of T-ALL was revealed and provided new insight into the molecular mechanisms that drive leukemogenesis and exposed new targets for treatment [2, 7, 8]. Over 70% of T-ALL patients carry gain-of-function mutations in transmembrane receptor NOTCH1, inducing ligand-independent activation or delayed degradation of intracellular NOTCH1. Alternatively, loss-of-function mutations of ubiquitin-ligase FBXW7 also cause delayed degradation of intracellular NOTCH1. The high prevalence of both mutations indicates the importance of NOTCH1 as a potential therapeutic target for the treatment of T-ALL [8]. Even in the presence of mutations, NOTCH1 activation requires cleavage of the transmembrane protein by the gamma-secretase complex for activation of signaling [9]. Therefore, signaling can be abrogated by inhibition of this catalytic complex with targeted inhibitors [10]. Although gamma-secretase inhibitors effectively inhibit proliferation of T-ALL in vitro and in vivo, clinical development is hampered by dose-limiting on-target induction of severe intestinal goblet cell hyperplasia as well as by their modest efficacy [11, 12]. The gamma-secretase complex contains either presenilin 1 (PSEN1) or presenilin 2 (PSEN2) as catalytic subunit. In T-ALL, the expression of PSEN2 is suppressed [13], allowing the targeted inhibition of PSEN1-containing complexes, thus avoiding PSEN2-mediated adverse effects [14]. Habets et al. [13] demonstrated that PSEN1-selective targeting of the gamma-secretase complex with MRK-560 is safe and effective for T-ALL. However, an urgent need for drugs that synergistically enhance the antileukemic effects of this class of molecules still remains [15].

The discovery of the BCR-ABL1 fusion kinase in chronic myeloid leukemia prompted the development of the targeted inhibitor imatinib, the first small molecule signal transduction inhibitor approved for clinical use, which greatly improved long-term survival for patients with chronic myeloid leukemia [16]. Similarly, the development of the JAK1/JAK2 kinase inhibitor ruxolitinib was driven by the high prevalence of mutations in JAK2 in myeloproliferative neoplasms. Ruxolitinib could be effective in about 20–30% of T-ALL cases with JAK-STAT pathway activation where JAK1 plays a central signaling role [17]. Similarly, imatinib could be used for rare T-ALL cases with ABL1 fusions, but case reports have shown variable efficacy in this setting [18]. Recently, selective inhibition of nuclear export by the second-generation exportin-1 (XPO1) inhibitor KPT-8602 (eltanexor) was shown to induce apoptosis in various cancer types, including ALL [19, 20], without deleterious effects to normal hematopoiesis [21]. Interestingly, the cancer-specific properties of this small molecule do not require specific mutations and previous studies in ALL have shown a general sensitivity of ALL cells for KPT-8602, independent of any genomic markers [19–21].

Here, we explored synergistic treatment combinations for NOTCH1 or FBXW7 mutated T-ALL in terms of efficacy and tolerance. In a first approach, we added ruxolitinib or imatinib in the presence of JAK3 mutations or the NUP214-ABL1-fusion, respectively. Our second aim was to expand combinatorial targeted therapy to the largest possible number of T-ALL cases by testing the synergy between MRK-560 and KPT-8602, a drug combination that could be effective in all T-ALL cases with activated NOTCH1 signaling. Our results show that the combination of PSEN1-selective gamma-secretase inhibition with a second targeted treatment is highly effective and does not cause additional toxicity.

## Methods

### Cell culture

ALL-SIL, HSB-2, LOUCY, HPB-ALL, DND41, RPMI-8402 and JURKAT cell lines were obtained from DSMZ and cultured in RPMI-1640 medium supplemented with 20% fetal bovine serum (Invitrogen, CA, USA) in 5% carbon dioxide at 37 °C.

*Compounds.* Dimethyl sulfoxide Hybri-Max (D2650), (2-Hydroxypropyl)-β-cyclodextrin (H107), Tween-80 (P1754), meglumine (M9179) and methylcellulose (M0262) were purchased from Sigma-Aldrich. MRK-560 was purchased from Tocris Bioscience. KPT-8602 (eltanexor) was bought from Selleckchem. Imatinib mesylate was obtained from AdooQ BioScience (A10468) and ruxolitinib phosphate from MedChem Express (HY-50858).

*Western blotting.* Cell lysates were prepared using 1X Cell Lysis Buffer (Cell Signaling) containing protease inhibitors (cOmplete—EDTA-free, Roche), PhosSTOP (Roche) and 5 mM Na3VO4. Proteins were separated by SDS-PAGE (NuPAGE NOVEX 4–12% Bis Tris, Invitrogen) and transferred to a nitrocellulose membrane using the Mini Trans-Blot Cell system (Bio-Rad). Labelling was carried out using unlabeled primary antibodies: Cleaved NOTCH1 (#2421, Cell Signaling Technology), PTEN (#9552, Cell Signaling Technology), β-actin (Sigma-Aldrich A1978). Western blot detection was performed with secondary antibodies conjugated with horse-radish peroxidase (GE Healthcare). Images were acquired using a cooled charge-coupled device camera system (ImageQuant LAS-4000, GE Health Care). For cleaved NOTCH1, cells were treated with MRK-560 1 µM or PF-03084014 1 µM for 24 h prior to lysis.

### Proliferation assays and inhibitor treatment

T-ALL cells were treated with DMSO, single compound or a combination of compounds for up to 14 days. Cells were seeded at a starting concentration of 3 × 10^5^ cells per mL and sub-cultured every 2–3 days by centrifugation, resuspension in fresh medium and addition of new compound. Cell concentration and viability were determined by FSC/SSC on a Guava EasyCyte Benchtop Flow cytometer (Merck Millipore). MRK-560 does not cause an immediate effect on cell proliferation and survival, and therefore, pretreatment was used when combined with other drugs in short-term experiments. Seven-day pretreated or untreated cells were seeded in 96-well plates (3 × 10^5^ cells/mL), and compounds (inhibitor or DMSO) were dispersed in a randomized fashion by a D300e digital dispenser (Tecan). Compound concentration was normalized to DMSO. A quantitative evaluation of proliferation was done after 48 h with ATPlite (PerkinElmer) and measured on the VICTOR X4 Reader (PerkinElmer). Synergy was analyzed using CompuSyn software.

*Apoptosis assay*. Apoptosis was measured after 12 days of treatment with DMSO, single compound or combination with the FITC Annexin V detection kit with PI (Biolegend). Cells were analyzed on a MACSQuant Vyb flow cytometer (Miltenyi Biotec), and data were analyzed using FlowJo Software (Becton, Dickinson and Company).

### Human primary leukemia samples

Primary leukemia samples were obtained at local institutions with informed consent. All experiments were conducted on protocols approved by the ethical committee of the University Hospital Leuven (UZ Leuven).

*Animal studies.* All mouse experiments were approved by the KU Leuven ethical committee and conducted according to EU legislation (Directive 2010/63/EU). NOD.Cg*-Prkdc*^*scid*^* Il2rg*^*tm1Wjl*^/SzJ (NSG) mice used for patient-derived xenograft experiments were bred in-house or purchased from Charles River Laboratories. During experiments, mice were housed in individually ventilated cages enriched with wood wool and shavings as bedding, with access to water and food *ad libitum* and monitored daily. Disease progression was monitored by weekly facial vein blood sampling followed by red blood cell lysis and staining with anti-hCD45-APC (#368512, BioLegend) for flow cytometry analysis (MACS Quant VYB, Miltenyi). After confirmation of engraftment, mice were randomized into treatment arms and treated according to protocol. For survival experiments, the presence of > 50% human CD45 + cells in the peripheral blood was used as surrogate end-point for leukemia-related death, and animals were subsequently euthanized (based on ethical guidelines). MRK-560 (30 μmol/kg) was dissolved in 20% hydroxypropyl-β-cyclodextrin (HP-β-CD) in 0.1 M meglumine for administration by intraperitoneal injection or 40% HP-β-CD in 0.3 M meglumine for oral gavage. KPT-8602 (5 mg/kg) was dissolved in 0.5% methylcellulose with 1% Tween-80 for oral gavage. Ruxolitinib (90 mg/kg) and imatinib (100 mg/kg) were dissolved in 0.5% methylcellulose and administered via oral gavage. In vivo treatment period was set at 2 weeks initially and extended to 3 weeks for later experiments to achieve a stronger response.

### Patient-derived xenografts

Primary cells were injected into the tail vein of 6- to 8-week-old female NSG mice. After successful engraftment, splenocytes were harvested and 1 × 10^6^ cells were injected into secondary recipient mice for treatment. Selected samples were transduced with the lentiviral pCH-SFFV-eGFP-T2A-fLuc vector and sorted for GFP-positivity using an S3e Cell Sorter (Bio-Rad) prior to reinjection into NSG mice.

### In vivo* bioluminescence imaging*

Mice were anesthetized with 2% isoflurane in 100% oxygen, injected subcutaneously with D-luciferin (126 mg/kg, Promega) dissolved in PBS (15 mg/mL) and placed in an IVIS Spectrum (Caliper Life Sciences) while maintaining anesthesia. Consecutive 2-min frames were acquired. Results are reported as maximum total flux per second for each whole mouse.

### Immunohistochemistry

After collection, tissues were fixed in 10% neutral buffered formalin (Sigma) for 48 h followed by processing for paraffin embedding (HistoStar Embedding Workstation). Sections of 7 µm thickness from the paraffin-embedded tissues (Thermo Scientific Microm HM355S microtome) were mounted on Superfrost Ultra Plus Adhesion slides (Thermo Scientific) and routinely stained with hematoxylin and eosin (Mayers Haematoxylin 1 l, 3801582E, Leica; Eosin Y solution, aqueous (1 L), HT110232-1L, Sigma-Aldrich) for histopathological examination. Then, sections were stained with periodic acid–Schiff (PAS). Slides were incubated in freshly prepared periodic acid (0.5%) for 15 min, rinsed, and incubated for 5 min in distilled water. The Schiff reagent (3952016-500ML, Sigma-Aldrich) was added onto the slides and kept for 15 min at room temperature in dark. The sections were then washed in slightly lukewarm running tap water for 5 min, rinsed and incubated in distilled water for 2 min, and counterstained in Mayer’s hematoxylin for 1 min. After washing in running tap water for 5 min, sections were dehydrated (95% EtOH, 100% EtOH, 100% EtOH, 3 min each, followed by two times xylene for 5 min) and mounted in DPX mounting medium (06,522, Sigma).

### Microscope image acquisition and image processing

Images were acquired on the Zeiss Axio Scan.Z1 using an × 20 objective and ZEN 2 software. For exporting images, the ZEN 2 software (Zeiss) was used. Analysis was performed with QuPath software.

### Statistical analyses

For analysis of synergy, the Chou-Talalay method was used, using CompuSyn software (ComboSyn, Inc). Graphs are presented as mean ± standard deviation unless stated otherwise, and all analyses were performed using GraphPad Prism. Comparison between two groups was made by the Student’s unpaired two-tailed *t*-test. One-way ANOVA with Bonferroni correction for multiple comparisons was used to examine differences when comparing effects in three or more groups. Survival in mouse experiments was represented with Kaplan–Meier survival curves, and statistical significance was calculated using the log-rank (Mantel Cox) test.

## Results

### Selective gamma-secretase inhibitor MRK-560 impairs cell growth in NOTCH1-dependent cells

**Fig. 1:**
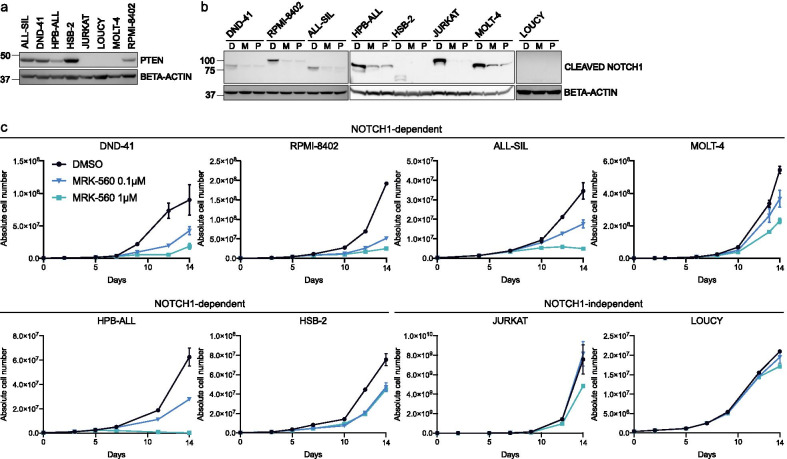
Selective gamma-secretase inhibitor MRK-560 inhibits cell growth in NOTCH1-dependent T-ALL cell lines. **a** Western blot analysis of PTEN protein levels in 8 T-ALL cell lines. Beta-actin was used as loading control. **b** Western blot analysis of cleaved NOTCH1 after treatment with DMSO (D), MRK-560 1 µM (M) or PF-03084014 1 µM (P) for 24 h. Beta-actin was used as loading control for all conditions; separate boxes represent separate blots. **c** Growth curves for DND-41, RPMI-8402, ALL-SIL, MOLT-4, HPB-ALL, HSB-2, JURKAT and LOUCY T-ALL cell lines. Growth medium was supplemented with DMSO, MRK-560 0.1 µM or MRK-560 1 µM. Data are shown as mean of biological triplicates. Standard deviation is depicted (only visible when larger than used symbols)

We tested the sensitivity to PSEN1-selective gamma-secretase inhibitor MRK-560 [22] in 6 NOTCH1-dependent and 2 NOTCH1-independent cell lines (JURKAT cells have a NOTCH1 mutation but are independent of NOTCH1 signaling due to AKT pathway activation and LOUCY has no NOTCH1 pathway mutations). Mutations in NOTCH1 and FBXW7 were confirmed using Sanger sequencing (Additional file 1: Table S1), and the presence of cleaved NOTCH1 and the PTEN protein was verified by Western blot analysis (Fig. 1a, b). In these cell lines, MRK-560 inhibited the formation of cleaved NOTCH1 to the same extent as the non-selective gamma-secretase inhibitor PF-03084014 [11, 23] (Fig. 1b). MRK-560 treatment caused a dose-dependent inhibition of proliferation/survival in cell lines that depend on NOTCH1 signaling (Fig. 1c). Together, these data indicate that MRK-560 is a potent inhibitor of NOTCH1 signaling with comparable activity as broad gamma-secretase inhibitors.

It has previously been suggested that FBXW7 mutations or PTEN loss can confer resistance to gamma-secretase inhibitors [24, 25]. However, treatment with MRK-560 strongly impaired cell growth of HPB-ALL and RPMI-8402 cells, which carry mutations in both NOTCH1 and FBXW7. Similar effects were observed for HSB-2 cells, a FBXW7 mutated and NOTCH1 wild-type cell line. Moreover, of the three cell lines that lack the PTEN protein, MOLT-4 was still sensitive to MRK-560. This supports previous observations that aberrations in FBXW7 or PTEN do not imperatively relieve T-ALL of its NOTCH1 addiction [26].

### Combining MRK-560 with tyrosine kinase inhibitors synergistically inhibits proliferation

**Fig. 2:**
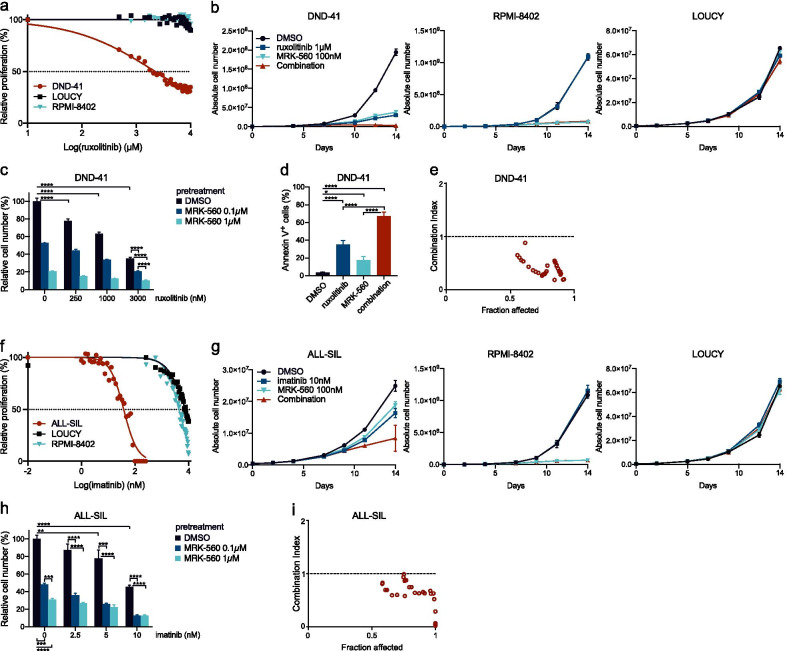
MRK-560 and tyrosine kinase inhibitors synergistically inhibit proliferation in vitro. **a** Dose–response curve for ruxolitinib treatment on DND-41, LOUCY and RPMI-8402 after 48-h incubation. Data represent mean of 3 experiments. **b** Growth curves for DND-41, RPMI-8402 and LOUCY T-ALL cell lines. Growth medium was supplemented with DMSO, MRK-560 100 nM, ruxolitinib 1 µM or both compounds. Data represent mean ± standard deviation for biological triplicates. **c** Bar chart showing relative cell number of DND-41 cells after 48-h incubation with different ruxolitinib concentrations following 7-day pretreatment with DMSO, MRK-560 0,1 µM or 1 µM. Bars represent mean, standard deviation is noted. Data are representative of biological triplicates. Data were analyzed using one-way ANOVA with Bonferroni multiple comparisons correction. **d** Annexin V-PI staining in DND41 T-ALL cells after 12 days of treatment with DMSO, ruxolitinib (1 µM), MRK-560 (1 µM) or combination. Data represent mean ± standard deviation for biological triplicates. Data were analyzed using one-way ANOVA with Bonferroni multiple comparisons correction. **e** Chou–Talalay plot for DND-41 showing the combined effect of MRK-560 and ruxolitinib after 48-h incubation. CompuSyn was used to calculate the combination index (CI). CI < 1, CI = 1 and CI > 1 indicates synergistic, additive and antagonistic effects, respectively. **f** Dose–response curve for imatinib treatment on ALL-SIL, LOUCY and RPMI-8402 after 48-h incubation. Data represent mean of 3 experiments. **g** Growth curves for ALL-SIL, RPMI-8402 and LOUCY T-ALL cell lines. Growth medium was supplemented with DMSO, MRK-560 100 nM, imatinib 10 nM or both compounds. Data represent mean ± standard deviation for biological triplicates. **h** Bar chart showing relative cell number of ALL-SIL cells after 48-h incubation with different concentrations of imatinib following 7-day pretreatment with DMSO, MRK-560 0,1 µM or 1 µM. Bars represent mean, standard deviation is marked. Data represent biological triplicates. Data were analyzed using one-way ANOVA with Bonferroni multiple comparisons correction. **i** Chou–Talalay plot showing the combined effect of MRK-560 and imatinib after 48-h incubation of ALL-SIL. CompuSyn was used to calculate the combination index (CI). **p < 0.01, ***p < 0.001, ****p < 0.0001

To test whether we could achieve synergistic activity when combining MRK-560 with other inhibitors, we focused on the genetic background of T-ALL. NOTCH1 and FBXW7 mutations are distributed evenly throughout the different genetic subgroups of T-ALL and, therefore, often co-occur with other targetable aberrations. Interleukin-7 (IL7) signaling activating mutations affect about one-third of T-ALL patients [8, 27] and occur in every component of the signaling pathway: the receptor α-chain IL7R, kinases JAK1 and JAK3 or the transcription factor STAT5 and the subsequent pathway activation can be inhibited by JAK-inhibitors [28, 29]. T-ALL cell line DND-41 depends on IL7 signaling due to a mutation in the IL7R gene, which is reflected in its sensitivity to the JAK kinase inhibitor ruxolitinib (Fig. 2a).

To examine the combined effect of MRK-560 and ruxolitinib, we cultured the cells for 14 days in medium supplemented with either MRK-560 (100 nM) or ruxolitinib (1 µM) alone or together. The drug combination induced a greater reduction in proliferation than single-drug treatment for DND-41, but not in the control cell lines RPMI-8402 and LOUCY, supporting the on-target effects of ruxolitinib and MRK-560 (Fig. 2b). Additionally, 7-day pretreatment with MRK-560 enhanced the efficacy of JAK kinase inhibition, yielding a significantly lower cell viability than with single-drug treatment (Fig. 2c) and increased apoptosis (Fig. 2d). Pretreatment with MRK-560 was used as this drug does not induce immediate effects on proliferation or survival. Calculation of combination indices (CI) revealed that the combination of both inhibitors was synergistic and affected a large fraction of the treated cells (Fig. 2e).

We applied a similar approach for fusions containing the ABL1 tyrosine kinase. NUP214-ABL1 is the most frequent ABL1 fusion gene in T-ALL (6% of T-ALL cases) [30] and other (rare) fusions are ETV6-ABL1, BCR-ABL1, ZBTB16-ABL1 and EML1-ABL1 [6, 31]. ALL-SIL carries the NUP214-ABL1 fusion, which makes it sensitive to ABL1-kinase inhibitor imatinib [5] (Fig. 2f). When cultured in the presence of MRK-560 (100 nM) and imatinib (10 nM), ALL-SIL showed a much greater proliferation deficit when both inhibitors were combined (Fig. 2g). Pretreatment of ALL-SIL with MRK-560 for 7 days strongly increased the effect of imatinib (Fig. 2h), even at low concentrations, and the nature of this drug cooperation was clearly synergistic (Fig. 2i).

### In vivo* combination of MRK-560 with tyrosine kinase inhibition reduces leukemic bone marrow infiltration*

**Fig. 3:**
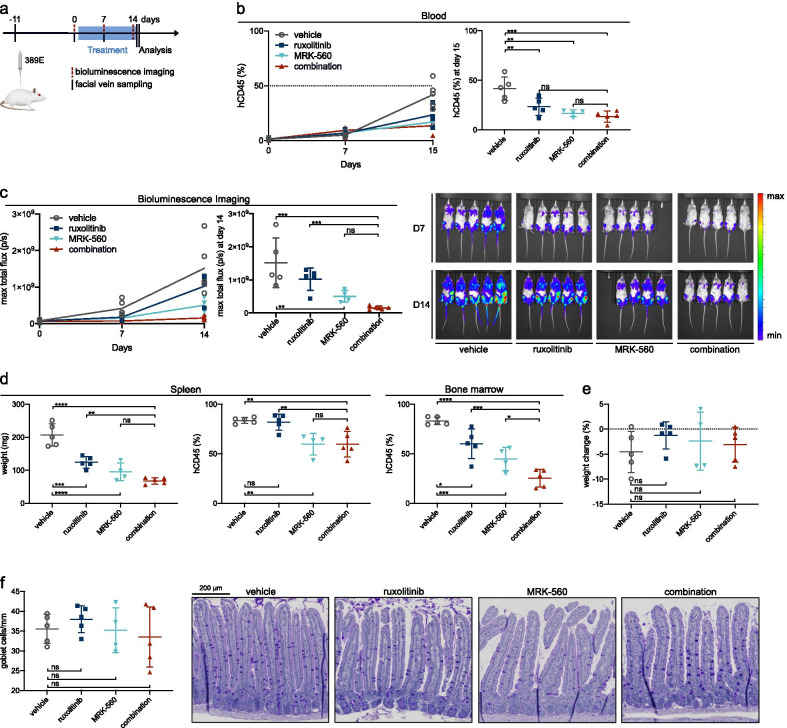
In vivo combination of MRK-560 with tyrosine kinase inhibition reduces leukemic bone marrow infiltration in a PDX model. **a** Schematic representation of the experiment. BLI = bioluminescence imaging. **b** Percentage of hCD45^+^ cells in the blood as assessed by flow cytometry analysis of hCD45-APC after facial vein blood sampling. Left panel depicts evolution; right panel shows analysis at the end of the experiment. Each dot represents a single animal; mean and standard deviation are shown. Data were analyzed by one-way ANOVA; Bonferroni correction for multiple comparisons testing was applied. **c** Maximum total flux (photons/second) for bioluminescence analysis for all mice. Evolution (left panel) and analysis at the end of the experiment (right panel) are shown. Each dot represents a single animal; mean and standard deviation are shown. Data were analyzed by one-way ANOVA; Bonferroni correction for multiple comparisons testing was applied. Normalized luminescence imaging overlay for all mice at day 7 and day 14 is shown on the right. **d** Spleen weight and hCD45 staining results for spleen and bone marrow on day 15. Each dot represents an animal; mean and standard deviation are shown. Data were analyzed by one-way ANOVA, and Bonferroni correction for multiple comparisons testing was applied. **e** Body weight change during treatment for each animal. Mean and standard deviation are shown. f) Quantification of number of goblet cells per millimeter of villus is shown on the left for each animal with mean and standard deviation. **g** Representative images of PAS staining from mice treated with vehicle, ruxolitinib, MRK-560 or combination for 14 days. Scale bar, 200 µm. ns = not significant (*p* > 0.05), *p < 0.05, **p < 0.01, ***p < 0.001, ****p < 0.0001

To verify and extend the preclinical value of our in vitro data, we set up in vivo validation experiments in a patient-derived xenograft (PDX) model. Patient sample 389E was derived from a T-ALL patient with mutations in NOTCH1 and JAK3 (Additional file 2: Table S2). The sample was injected into 20 NOD.Cg-Prkdc^scid^ Il2rg^tm1Wjl^/SzJ (NSG) immunodeficient mice via tail vein injection. After engraftment, mice were randomized to vehicle, MRK-560 [30 µmol/kg, intraperitoneal injection (IP)], ruxolitinib [90 mg/kg, oral gavage (PO)] or combination treatment and were treated for 14 days (Fig. 3a). Although leukemia progression was effectively reduced in all treatment conditions when assessed by blood count of human CD45^+^ (hCD45^+^) cells, the combination arm did not have significantly less leukemic cells in the blood after 14 days of treatment (Fig. 3b). However, when global disease burden was measured using in vivo bioluminescence imaging (BLI), the combination outperformed ruxolitinib-only treatment (Fig. 3c). Analysis of the mice after the 2-week treatment period revealed a significantly smaller spleen size, associated with reduced leukemic infiltration (Fig. 3d), for all treatment groups when compared to vehicle-treated mice. Adding MRK-560 to ruxolitinib clearly increased the efficacy of JAK-inhibition, but results were not better than MRK-560 alone. In contrast, the bone marrow was significantly less infiltrated by leukemic cells in the combination treatment. Importantly, no signs of increased gastrointestinal toxicity were noted in the mice. We did not observe pathological changes in the gut architecture. The abundance of goblet cells was assessed by periodic acid-Schiff (PAS) staining and was unaltered by the treatment, resulting in similar weight changes for all treatment groups (Fig. 3e, f).

### In vivo* sensitivity to PSEN1-selective gamma-secretase inhibition of an FBXW7 mutated PDX is enhanced by addition of imatinib*

**Fig. 4:**
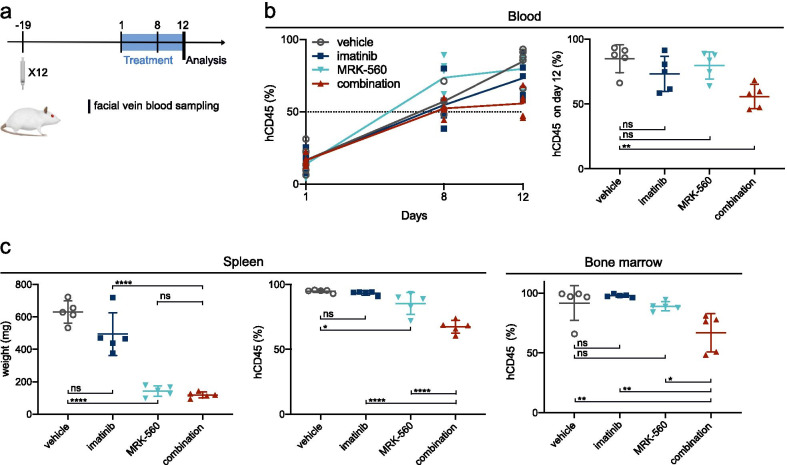
In vivo sensitivity to NOTCH1-inhibition of a FBXW7 mutated PDX is enhanced by association of imatinib. **a** Schematic representation of the experiment. **b** Percentage of hCD45^+^ cells in the blood as assessed by flow cytometry analysis of hCD45-APC after facial vein blood sampling. Evolution is shown on the left; analysis on day 12 is shown on the right. Each dot represents a single animal; mean and standard deviation are shown. Data were analyzed by one-way ANOVA; Bonferroni correction for multiple comparisons testing was applied. **c** Spleen weight and hCD45 staining results for spleen and bone marrow on day 12. Each dot represents an animal; mean and standard deviation are shown. Data were analyzed by one-way ANOVA, and Bonferroni correction for multiple comparisons testing was applied. ns = not significant (*p* > 0.05), *p < 0.05, **p < 0.01, ****p < 0.0001

We showed that, in vitro, FBXW7-mutated T-ALL can be sensitive to gamma-secretase inhibition (Fig. 1c). To test this in vivo, we used PDX sample X12, which contains an inactivating mutation in FBXW7 and a NUP214-ABL1 fusion gene. After engraftment into NSG mice, animals were randomized and treated with MRK-560 (30 µg/kg, PO) and/or imatinib (100 mg/kg, PO) or vehicle (Fig. 4a). Due to the aggressive nature of this sample, mice could only be treated for 12 days after confirmed engraftment before vehicle-treated animals needed to be euthanized. After 7 days of treatment, disease progression was slowed down in treatment groups receiving MRK-560, which supports in vivo sensitivity of FBXW7-mutated T-ALL to NOTCH1 inhibition. Despite the short treatment duration, hCD45^+^ blood counts were significantly reduced in the mice that received the combination treatment (Fig. 4b). Treatment with MRK-560 reduced spleen size, and the addition of imatinib significantly enhanced the reduction in spleen infiltration compared to MRK-560 alone (Fig. 4c). This superior effect on tissue infiltration was reflected in the bone marrow, where only the combination of MRK-560 and imatinib effectively reduced the leukemia burden.

### PSEN1-selective gamma-secretase inhibition synergizes with nuclear export inhibition in vitro

**Fig. 5:**
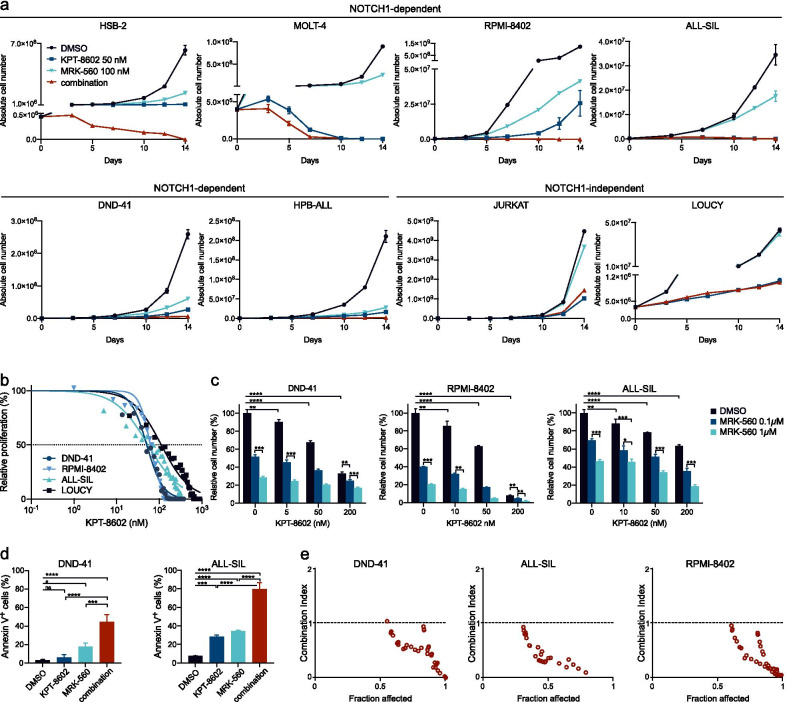
NOTCH1-inhibition synergizes with nuclear export inhibition in vitro. **a** Growth curves for HSB-2, MOLT4, RPMI-8402, ALL-SIL, DND-41, HPB-ALL, JURKAT and LOUCY cell lines in the presence of DMSO, KPT-8602 50 nM, MRK-560 0.1 µM or both inhibitors. Data are shown as mean of biological triplicates. Standard deviation is depicted only when larger than the used symbol. **b** Dose–response curve for KPT-8602 for DND-41, RPMI-8402, ALL-SIL and LOUCY. Data represent the mean of biological triplicates for any given concentration. **c** Bar charts showing cell numbers for DND-41, RPMI-8402 and ALL-SIL relative to control for different concentrations of KPT-8602 after 7-day pretreatment with DMSO, MRK-560 0.1 or 1 µM. Data are shown as mean ± standard deviation of biological triplicates. Significance was calculated using one-way ANOVA with Bonferroni’s multiple comparisons correction. **d** Annexin V-PI staining in DND41 and ALLSIL cells after 12 days of treatment with DMSO, KPT-8602 (50 nM), MRK-560 (1 µM) or combination. Data represent mean ± standard deviation for biological triplicates. Data were analyzed using one-way ANOVA with Bonferroni multiple comparisons correction. **e** Combination index plots according to Chou-Talalay. Each dot represents a concentration combination. ns = not significant (*p* > 0.05), *p < 0.05, **p < 0.01, ****p < 0.0001

Because selective inhibitors of nuclear export do not require a mutated target and were shown to inhibit the growth of all subtypes of ALL, combination of KPT-8602 and MRK-560 could potentially benefit all T-ALL patients with activated NOTCH1 signaling. To test whether MRK-560 and KPT-8602 act synergistically, we cultured T-ALL cell lines for 14 days in the presence of DMSO, MRK-560 (100 nM), KPT-8602 (50 nM) or both inhibitors (Fig. 5a). All cell lines were sensitive to KPT-8602, independent of NOTCH1 or FBXW7 status. For NOTCH1-dependent cells, the combination of both inhibitors was highly effective, strongly inhibiting leukemia proliferation and/or inducing cell death. This could not be achieved with single-drug treatment, except in MOLT-4 where the combination accelerated the effect of KPT-8602. We selected 3 cell lines with similar sensitivity to KPT-8602 (Fig. 5b), to study the cooperation between these compounds. Seven days of pretreatment with MRK-560 increased sensitivity to KPT-8602, resulting in significantly lower proliferation/viability (Fig. 5c) and increased apoptosis (Fig. 5d). For the majority of the tested concentrations, the combination of MRK-560 with KPT-8602 was synergistic (CI < 1) (Fig. 5e).

### *Leukemia burden is reduced *in vivo* when MRK-560 and KPT-8602 are combined*

**Fig. 6:**
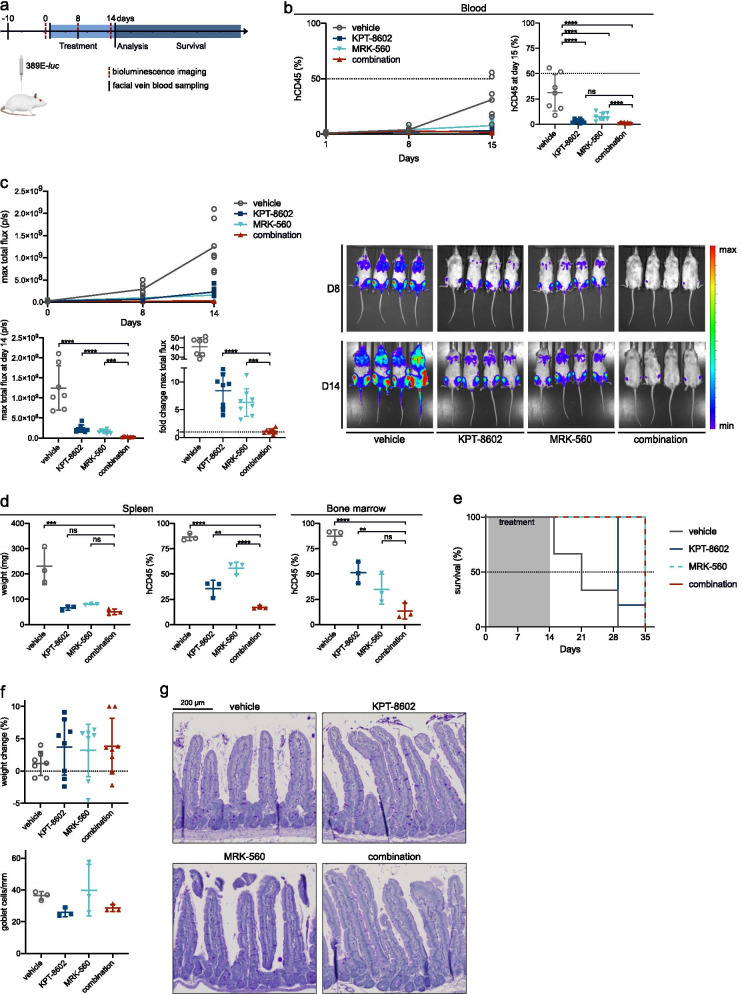
Combination of MRK-560 and KPT-8602 reduces leukemia burden in vivo. **a** Schematic representation of the experiment. **b** Percentage of hCD45^+^ cells in the blood as assessed by flow cytometry analysis of hCD45-APC after facial vein blood collection over time (left) and at day 15 (right). Each dot represents a single animal; mean and standard deviation are shown at day 15. Data were analyzed by one-way ANOVA; Bonferroni correction for multiple comparisons testing was applied. **c** Bioluminescence imaging results for all mice, measured as maximum total flux (photons/second). Evolution over time (top) and analysis at day 14 (bottom, left) of the experiment are shown. Each dot represents a single animal; bars represent mean and standard deviation. Fold change in maximum total flux between end and start of treatment was calculated for each mouse (bottom, middle). Data were analyzed by one-way ANOVA; Bonferroni correction for multiple comparisons testing was applied. Normalized luminescence imaging overlay for all mice at days 0, 7, and day 14 is shown on the right panel. **d** Spleen weight and hCD45 staining results for spleen and bone marrow on day 15. Each dot represents an animal; mean and standard deviation are shown. Data were analyzed by one-way ANOVA, and Bonferroni correction for multiple comparisons testing was applied. **e** Kaplan–Meier plot showing survival for the remaining 5 mice in every treatment group. Gray area indicates treatment period. **f** Body weight change during treatment for each animal (top) and quantification of number of goblet cells per millimeter of villus (bottom) (*n* = 3). Mean and standard deviation are shown. **g** Representative images of PAS staining from mice treated with vehicle, KPT-8602, MRK-560 or combination for 14 days. Scale bar, 200 µm. ns = not significant (*p* > 0.05), *p < 0.05, **p < 0.01, ***p < 0.001, ****p < 0.0001

For in vivo validation, we injected 32 NSG mice with NOTCH1- and JAK3-mutated PDX sample 389E. After engraftment and randomization, mice were treated for 14 days with vehicle, KPT-8602 (5 mg/kg, PO), MRK-560 (30 µg/kg, IP) or both compounds (Fig. 6a). hCD45^+^ lymphoblasts in the blood were effectively reduced in both single-drug treatment arms and nearly eradicated in the animals treated with the combination (Fig. 6b). The difference between KPT-8602-only treatment and combination did not reach significance in the blood. However, systemic evaluation of leukemia burden by bioluminescence imaging clearly showed superiority of combination treatment (Fig. 6c). Single-drug treatment significantly slowed down leukemia development, but to a lesser extent than combination treatment. After 14 days of treatment, 50% of the mice in the combination condition had maximum total flux values below their starting value, as represented by the fold change < 1 (range 0.52–1.93). Subsequently, 3 mice were randomly selected from every group and were euthanized for tissue analysis. None of the treated mice developed splenomegaly, but the mice from the combination arm showed significantly less leukemic infiltration in the spleen, when compared to single-treatment arms (Fig. 6d). Bone marrow disease was reduced, but the difference in bone marrow infiltration was not significant. As a consequence, combination treatment did not yield a survival benefit (Fig. 6e). None of the mice developed goblet cell hyperplasia or significant weight loss (Fig. 6f, g).

### Combined inhibition of PSEN1-gamma-secretase and nuclear export prolongs survival in T-ALL

**Fig. 7:**
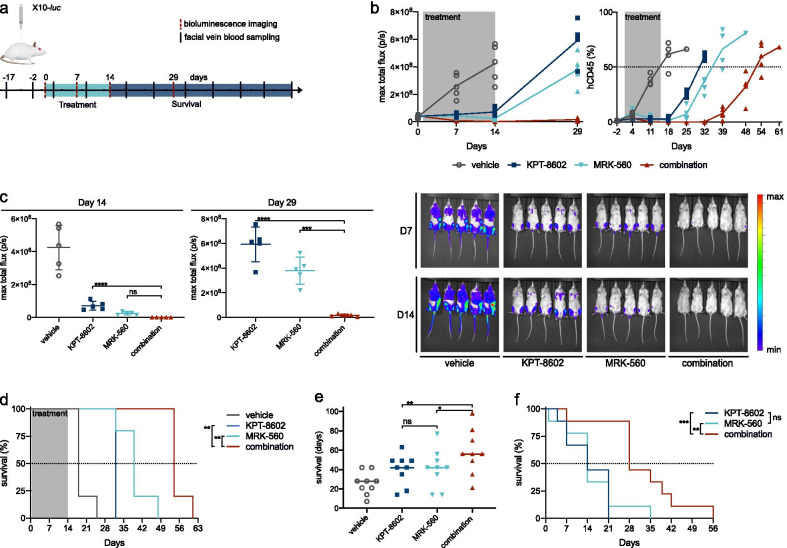
Combined inhibition of NOTCH1 and nuclear export prolongs survival in T-ALL. **a** Schematic representation of the experiment. **b** Evolution of bioluminescence signal (left) and percentage of hCD45^+^ cells in peripheral blood (right) over time, every dot represents an animal. Grey area represents the treatment period. **c** Bioluminescence analysis measured by maximum total flux (p/s) at day 14 and day 29 for each mouse; mean and standard deviation are noted. Bioluminescence imaging overlays are (right panel) shown for reference. Data were analyzed by one-way ANOVA with Bonferroni correction for multiple comparisons. **d** Kaplan–Meier survival plots for X10-*luc* mice. Data were analyzed using the log-rank Mantel–Cox test. **e** Scatter dot plot of survival in days from start of treatment for each mouse. Line represents median for each treatment group. Data were analyzed using one-way ANOVA for matched data with Bonferroni multiple comparisons correction. **f** Kaplan–Meier plot for survival, starting from death of vehicle. Data were analyzed using the log-rank Mantel–Cox test. ns = not significant (*p* > 0.05), *p < 0.05, **p < 0.01, ***p < 0.001, ****p < 0.0001

To study whether the observed reduction in measurable leukemic disease could improve survival nonetheless, we set up a survival experiment with patient sample X10-luc. Twenty NSG mice were randomized and treated for 14 days with the same regimen as described for 389E (Fig. 7a). Disease progression and efficacy of treatment were followed by BLI and measurement of hCD45 in peripheral blood (Fig. 7b, c). The presence of > 50% hCD45^+^ cells in the blood was considered as surrogate endpoint for death due to leukemia. At the end of the 14-day treatment period, global disease burden as measured by bioluminescence was strongly reduced in all treated mice, without a significant benefit of combination treatment over MRK-560 monotherapy (Fig. 7c). Nevertheless, when treatment was stopped, we noted an early flare-up of the disease in the animals from the single-drug treatment arms, and by days 29 and 32, respectively, the bioluminescence and hCD45^+^ levels had risen to those of untreated mice. In contrast, in the mice receiving the combination treatment, the disease remained suppressed, and at day 29 (BLI) or day 32 (blood analysis) disease levels were still lower than at the start of the treatment (Fig. 7b, c). This prolonged suppression of T-ALL resulted in a significantly longer survival for the animals in the combination group when compared to MRK-560 (1.4-fold, *p* = 0.002) and KPT-8602 (1.69-fold, *p* = 0.0027) (Fig. 7d).

To evaluate whether these results could be confirmed in a broader cohort of NOTCH1- or FBXW7-mutated T-ALL patients, we set up a drug treatment trial with nine different PDX samples. Two samples (XD83 and XD83q) were a matched diagnosis—relapse pair derived from the same patient. Each sample was injected into four NSG mice, and mice were randomized so that every treatment group contained 1 mouse for every PDX sample. Mutations for all PDX’s were verified by Sanger sequencing and are listed in Additional file 2: Table S2. This approach allowed us to run a small-scale clinical trial-like experiment to validate the predicted survival benefit in a cohort of PDX samples, each with its own mutational background and proliferation rate.

Because of the delayed effect of MRK-560 (Fig. 4b, 7b) on hCD45^+^ blood levels in some of the treated mice in earlier experiments, we used here a treatment period of 3 weeks (5 days on, 2 days off treatment). Blood was sampled every week, and the presence of more than 50% hCD45^+^ cells was considered as surrogate endpoint for survival. Median survival was 28 days for vehicle treated mice, 42 days for mice that received single-drug treatment and 56 days for the mice in the combination treatment arm. Mice in all treatment arms lived longer than vehicle-treated mice. Four out of 9 vehicle-treated mice succumbed during the treatment, as compared to 2 mice for single-drug treatment and 1 for the combination group, reflecting the aggressiveness of the selected PDX samples and supporting both the efficacy and tolerability of the treatment regimen.

We observed no difference in survival between the two single-drug groups (*p* > 0.9999). However, the mice that were treated with both compounds together lived significantly longer than those treated with MRK-560 only (*p* = 0.0142) or KPT-8602-only (*p* = 0.0058) (Fig. 7e). As expected, a large variability in the rate of disease progression was observed between the different PDX samples with survival ranging from 7 to 42 days from the start of treatment for the vehicle-treated mice. Therefore, we plotted survival as the number of days that mice in the treatment groups outlived their vehicle-treated counterpart (Fig. 7f). Survival curves for KPT-8602 and MRK-560 treated mice were similar (*p* = 0.856), and both treatment groups outlived their vehicle-treated littermate with a median of 14 days. This survival benefit was twice as long in the combination group, resulting in a clear separation between survival curves (*p* = 0.0005 vs KPT-8602; *p* = 0.0049 vs MRK-560), supporting the added benefit of combining nuclear export inhibition with gamma-secretase inhibition in T-ALL.

## Discussion

It is unlikely that a single molecule will be able to cure T-ALL. Combining inhibitors could improve efficacy, if done in a rational manner. Targeting NOTCH1 signaling offers the opportunity to target about 70% of T-ALL patients, without subtype restriction [8]. Different approaches for successful NOTCH1 inhibition are under evaluation: interference with NOTCH1 maturation by SERCA-inhibition, NOTCH-targeting inhibitory antibodies and more conventional targeted inhibitors. For the latter, very few gamma-secretase inhibitors have progressed past early-phase clinical studies [11, 12, 32], and none have made it to clinical practice because of dose-limiting toxicity or lack of clinical benefit. As a consequence, there are currently no clinical trials with a gamma-secretase inhibitor ongoing for T-ALL.

Here, we present an extensive preclinical characterization of the PSEN1-selective gamma-secretase inhibitor MRK-560. MRK-560 potently inhibits the proliferation of lymphoblasts both in vitro and in vivo in PDX models. Moreover, we show that MRK-560 reduces leukemic tissue infiltration and prolongs survival without the characteristic gastrointestinal toxicity of non-selective gamma-secretase inhibitors. We also show that sensitivity to MRK-560 is not limited to NOTCH1 mutated cases but can extend to patients carrying mutations in FBXW7.

To increase efficacy, we combined MRK-560 with imatinib or ruxolitinib, inhibitors with a long-standing experience in clinical practice. We show that the combination of MRK-560 with these inhibitors is synergistic and effectively counters leukemia progression and reduces tissue infiltration in vivo. Importantly, even in short-term experiments, combination treatment provided a clear benefit in the bone marrow niche, suggesting a protective effect against relapse. Finally, the strongest effect was noted in the combination of MRK-560 with nuclear export inhibitor KPT-8602, which makes it possible to obtain the maximum treatment effect in the largest possible cohort, since the majority of T-ALL cases harbor NOTCH1 pathway mutations and no specific mutations are required for sensitivity toward KPT-8602. The association of these two compounds provided a survival benefit across different genetic backgrounds in our PDX models. Although our treatment experiments are limited in terms of animal numbers, the results are consistent over the different genetic backgrounds of the PDX samples. We provide substantial preclinical data to support the development and validation of PSEN1-selective gamma-secretase inhibitors for the treatment of a wide range of T-ALL patients. To conclude, we show that the antileukemic effect of gamma-secretase inhibition can be safely maximized by well-orchestrated combination of small molecule inhibitors providing the rationale for further investigation of PSEN1-selective gamma-secretase inhibitor-based treatment regimens.

## Supplementary Information

**Additional file 1.** Supplemental Table 1 describing the cell lines used.

**Additional file 2.** Supplemental Table 2 describing the PDX models used.

## Data Availability

Cell lines used in this study are commercially available from www.dsmz.de. PDX samples used in this study can be obtained upon request. No datasets were generated in this study.

## Abbreviations

BLI: Bioluminescence imaging
PAS: Periodic acid–Schiff
PDX: Patient-derived xenograft
T-ALL: T cell acute lymphoblastic leukemia
